# Phacomatosis pigmentovascularis of cesioflammea type*

**DOI:** 10.1590/abd1806-4841.20164516

**Published:** 2016

**Authors:** Delky Johanna Villarreal Villarreal, Fabiano Leal

**Affiliations:** 1 Instituto de Dermatologia Professor Rubem David Azulay – Santa Casa da Misericórdia do Rio de Janeiro (IDPRDA - SCMRJ) – Rio de Janeiro (RJ), Brazil; 2 Hospital Naval Marcílio Dias (HNMD) – Rio de Janeiro (RJ), Brazil

**Keywords:** Melanocytes, Nevus, Nevus of Ota, Port-wine stain, Skin abnormalities, Skin pigmentation

## Abstract

Phacomatosis pigmentovascularis is a rare syndrome, defined as the simultaneous presence of vascular nevus and melanocytic nevus in the same patient. We report the case of a 53-year-old woman presented with dermal melanosis and extensive vascular nevus, which match the typical manifestations of phakomatosis pigmetovascularis of cesioflammea type, according to Happle's classification. The rare occurrence of this genodermatosis and the clinical exuberance of the skin lesions motivated this case report.

## INTRODUCTION

Phakomatosis pigmentovascularis is a rare, sporadic genetic syndrome characterized by the occurrence of vascular and pigmented nevi with or without extracutaneous manifestations.^1,2^ 247 cases have been reported in the literature so far, the vast majority of them in Japan.^1,3,4^ We report a case of phakomatosis pigmentovascularis of cesioflammea type with bulbar melanosis as the only extracutaneous manifestation.

## CASE REPORT

**Figure 1:**
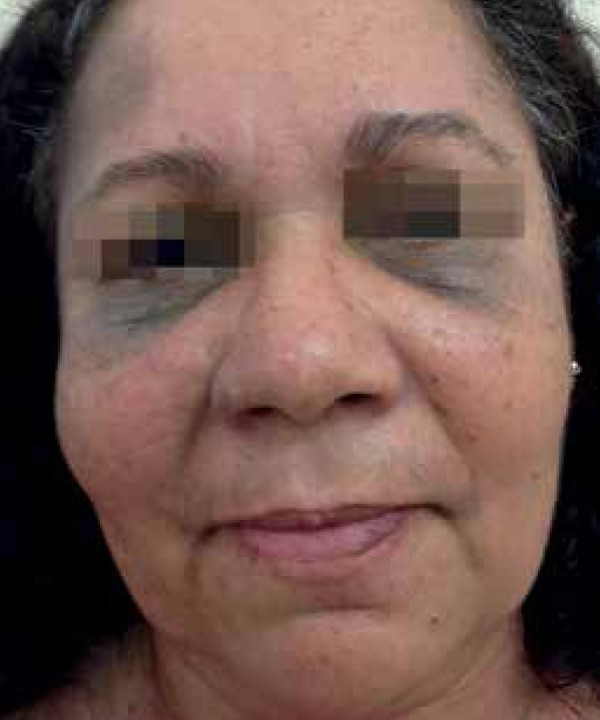
Nevus of Ota

**Figure 2:**
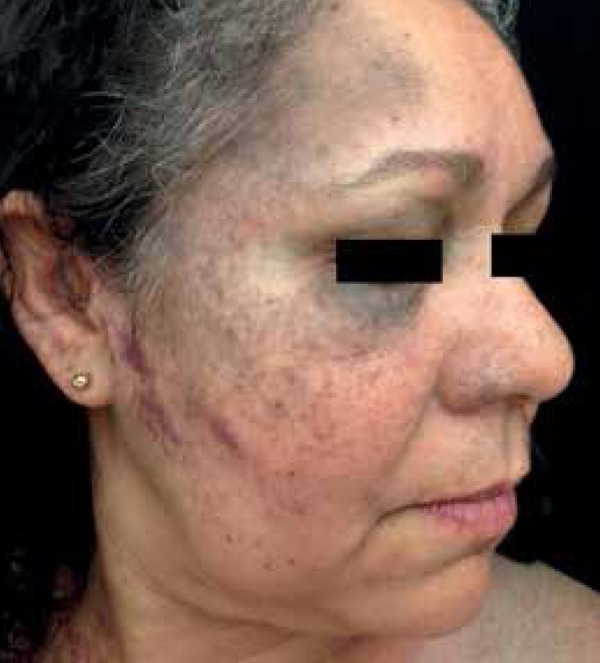
Nevus of Ota Blue-grayish spots of speckled appearance on the forehead and right malar

**Figure 3:**
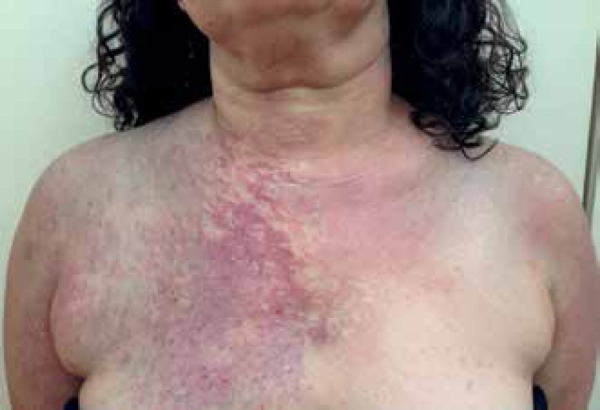
Nevus of Ito, interspersed with Port-wine stains, and anemic nevus on her lap

**Figure 4:**
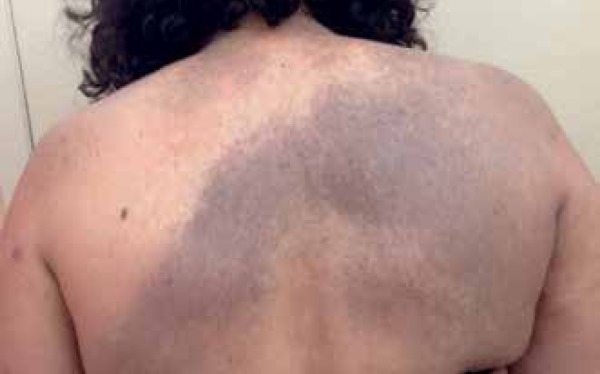
Nevus of Ito, Blue-grayish macule with stained aspect

A 53-year-old female patient from Rio de Janeiro was referred to our hospital with the following manifestations from birth: 1) blue-grayish spots with speckled appearance on her face, chest and back, suggesting nevus of Ota and Ito (Figures 1 to 4); 2) port-wine stains interspersed with anemic nevus in the right preauricular region, on the anterior thorax, and right upper limb (Figure 3); and 3) melanosis on her right eyelid. All lesions were present at birth and increased in size after pregnancy. Physical and neurological examination showed normal nails, mucous membrane, hair, and legs. She denied significant family history and parental consanguinity.

## DISCUSSION

The term phakomatosis originally had a wider meaning, as it was used to describe some neurocutaneous syndromes such as neurofibromatosis, tuberous sclerosis, and phakomatosis pigmentovascularis. However, it currently defines a group of genetic skin diseases characterized by the presence of two or more different kinds of nevi, with or without systemic involvement.

Phakomatosis pigmentovascularis (PPV), first described by Ota in 1947, is defined as the sporadic occurrence of vascular nevi associated with pigmented nevi. 247 cases have been reported in the literature, most of them in Japan. PPV of cesioflammea type (type II) is the most common.^1,3,4^

PPV was originally classified in 5 major subtypes (I -V) with additional categorization “a” (for isolated skin lesions) and “b” (for extracutaneous lesions).

1. Type I, capillary malformation (CM) associated with epidermal nevus;
2. Type II, CM associated with dermal melanosis, with or without anemic nevus;
3. Type III, CM associated with nevus spilus, with or without anemic nevus;
4. Type IV, CM, dermal melanosis and nevus spilus, with or without anemic nevus;
5. Type V, Cutis marmorata telangiectasia congenita associated with dermal melanosis.

In 2005, Rudolph Happle proposed a more practical and understandable classification model and described four types of PPV: ^5^

1. Phakomatosis cesioflammea (type IIa and IIb)
2. Phakomatosis Spilorosea (type IIIa and IIIb)
3. Unclassifiable forms of phakomatosis (type IV)
4. Phakomatosis cesiomarmorata (type V)

Happle’s classification abolished type I because the epidermal nevi do not originate from nevus cells, and, therefore, the term pigmented phakomatosis is innacurate. The author also classified as “unclassifiable forms of phakomatosis” some cases that could not be attributed to a well-defined clinical and genetic entity, of which phakomatosis type IV is part.^5^

PPV pathogenesis is still unclear, but it is believed to be an abnormality in the development of melanocytic nevus cells and vasomotor neural cells derived from the neural crest. PPV could be explained by a genetic phenomenon called twin spots or didymosis, a specific mechanism of somatic mosaicism. According to this phenomenon, abnormalities in two different cell lines (melanocytes and vasomotor nerve cells) suggest that the autosomal recessive mutations occur in two different loci. However, they are neighbors, located in the same chromosome region, and are simultaneously switched by somatic recombination, yielding two cloned cells with different phenotypes on a background of normal cells. Two adjacent areas of skin lesions eventually develop, with tissue mutants that differ from the circumjacent normal tissue.^1,6-9^

We classified our patient as type IIb PPV or phakomatosis cesioflammea based on the following clinical criteria: nevus of Ota, nevus of Ito and bulbar melanosis (the only extracutaneous manifestation), concomitant with Port-wine stains interspersed with anemic nevus without systemic involvement.

The word cesioflammea is a Latin-derived compound noun: *Caesius,* meaning blue-grayis, and *flammea,* which means fire or flame; therefore, PPV of cesioflammea type is characterized by the coexistence of blue spots (dermal melanosis) and capillary malformations.^1,2^ Other systemic or cutaneous abnormalities may be associated with phakomatosis cesioflammea, such as anemic nevus, alopecia, lipohypoplasia, and lower limb asymmetry. Glaucoma, dysplasia in veins and lymphatics, and syndromes such as Sturge-Weber and Klippel-Trenaunay, may come with this subtype of phakomatosis.^10^

The different PPV types without systemic involvement have a benign course and need no specific treatment. In some cases, due to aesthetic impact, lasers such as intense pulsed light and Q-switched have been studied for the treatment of pigmented nevi and nevi flammeus, respectively, with good results.^10^
